# Two Cases of Paraquat Poisoning from Kota, Rajasthan, INDIA

**DOI:** 10.1155/2012/652146

**Published:** 2012-12-03

**Authors:** Surendra Khosya, Sunil Gothwal

**Affiliations:** ^1^Rajasthan University of Health Sciences, Jaipur, India; ^2^Maharaja Agrasen Institute of Medical Education and Research, Agroha 125047, Haryana, India

## Abstract

Paraquat (1,1′-dimethyl-4,4′-dipyridylium) is a broad spectrum liquid herbicide associated with both accidental and intentional ingestion, leading to severe and often fatal toxicity. Despite widespread availability, reports of herbicide poisoning from India are not common. Diagnosis is often difficult in the absence of proper history, nonspecific clinical features, and lack of diagnostic tests. We report two cases of fatal paraquat poisoning from a tertiary care hospital, Kota, Rajasthan, India.

## 1. Introduction

Paraquat is a quaternary nitrogen herbicide that is sprayed on unwanted weeds and other vegetations before planting crops. It is a fast-acting, nonselective compound, which destroys tissues of green plants on contact and by translocation within the plant. Paraquat exerts its herbicidal activity by inhibiting reduction of NADP to NADPH during photosynthesis. This disruption leads to the formation of superoxide anion, singlet oxygen, and hydroxyl and peroxyl radicals. These reactive oxygen species (ROS) interact with the unsaturated lipids of membranes, resulting in the destruction of plant organelles, inevitably leading to cell death [1]. It is produced commercially as a brownish concentrated liquid of the dichloride salt in 10–30% strength under the trade name of “Gramoxone” and for horticultural use as brown granules called “Weedol” at about 5% concentration [2]. Paraquat poisoning has been widely reported worldwide, but only a few case reports are described in literature from India [3–5].

We are reporting two cases of fatal paraquat poisoning from north Indian state of Rajasthan. Both patients were admitted in a tertiary care hospital. They were agriculturists and belonged to area of the state where “Gramoxone” is freely available for its use in the fields as herbicide. On extensive literature search no case of paraquat poisoning was found to be reported from this state of India.

## 2. Case Report

### 2.1. Case Number 1

A previously healthy 34-year-old farmer was brought to the emergency department 1 h after ingestion of an unknown quantity of a liquid. The mother of the patient described the liquid as possibly a herbicide containing 2,4-D as the active ingredient. Following the ingestion, he had several episodes of vomiting. Relatives denied any episode of seizure. On examination, he was drowsy but arousable, febrile (101.5°F) with heart rate 110/min, regular, BP 110/86 mmHg, respiratory rate 20/min, and oxygen saturation (while breathing room air) 95%. His oral mucosa was congested and edematous. Pupils were bilateral 2 mm and reacting to light. Both lung fields were clear on auscultation. Rest of the systemic examination was unremarkable. 

**Figure 1:**
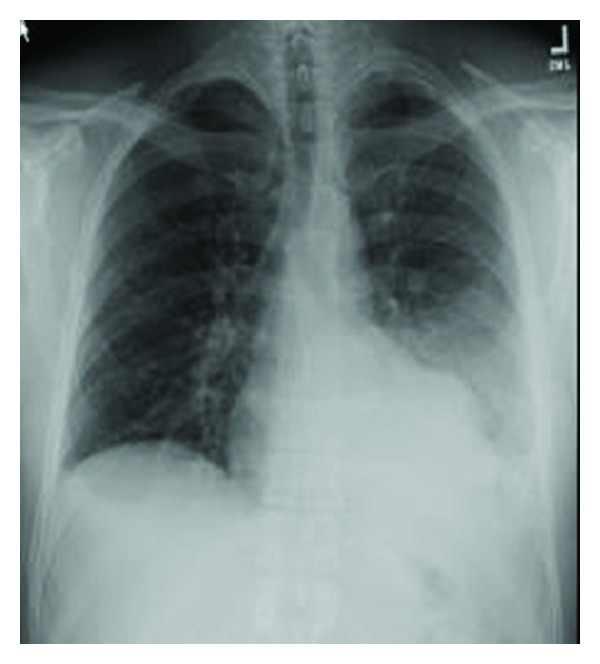
Chest X-ray on day 1 of patient 1.

Gastric lavage was performed, and repeated doses of charcoal were given in the emergency department, and he was admitted to the intensive care unit (ICU) for observation and further evaluation. In the ICU, he received IV fluid and antiemetic (Ondansetron) as supportive measure. Initial complete blood count, electrolytes, liver and renal function tests, arterial blood gas, serum amylase, and choline esterase levels were within normal limit. ECG revealed sinus tachycardia, and 2D echocardiography showed normal cardiac chambers with LVEF of 60%. His initial chest X-ray showed left lower zone infiltrate (Figure 1).

Eight hours after admission, the patient became severely hypoxic, SpO_2_ 89% while on 60% Venturi Mask was used. ABG showed PaO_2_ of 47 mmHg with mild respiratory alkalosis. Rapid sequence intubation was performed, and he was placed on mechanical ventilation. Fresh and altered blood was aspirated through the Ryle's tube. A probable diagnosis of pulmonary aspiration was made, and he was started on Piperacillin-Tazobactam and Clindamycin empirically. Pantoprazole infusion was started. On the following day, he continued to remain febrile. Blood investigations on day 2 were unremarkable except mild rise in BUN and serum creatinine (26 and 1.5 mg/dL). His arterial blood gas values on day 2 were pH 7.42, PaCO_2_ 34 mmHg, and PaO_2_ 64 mmHg (50% FIO_2_ and PEEP 5 cm H_2_O). There was no fresh change in the chest X-ray. On day 3, he remained febrile and drowsy. His FIO_2_ requirement decreased to 40%. On subsequent days in the ICU, he showed progressive improvement and became afebrile on day 4, and azotemia improved from day 3 onward. On day 6, he was conscious, obeying command, and had a PaO_2_/FIO_2_ ratio >300 with normal blood chemistry. He was extubated later on during the day. Subsequently, he revealed that the liquid that he had ingested is Gramoxone 24% that contains paraquat. The next day, he was transferred to the medical ward A, and 4 days later he was discharged following psychiatry consultation.

### 2.2. Case Number 2

A thirty-one-year-old female patient was admitted in the female medical ward A with history of ingestion of 20 mL of paraquat dichloride (Gramoxone-24% SL). She was having history of oliguria since last two days. On examination, she had tachycardia and was tachypnoeic. Oral erosions and icterus were present. On nervous system examination, deep tendon reflexes were absent. Rest of the examination was normal. The serum urea was 243 mg/dL, and creatinine was 10.5 mg/dL. Total serum bilirubin was 7.2 mg%, and conjugated was 4.2 mg%. The transaminases were raised (SGOT-189 IU and SGPT-210 IU). The alkaline phosphatase was 217 KAU. Patient was managed conservatively for two days including one session of haemodialysis and became hemodynamically unstable with features of adult respiratory distress syndrome. She died 2nd day despite of ventilatory support. 

## 3. Discussion

The present cases posed a number of diagnostic challenges to the critical care team. Firstly, there was considerable confusion regarding the identity of the liquid ingested. 2,4-D was sprayed in the field on the day of ingestion, so initially it was suspected as the ingested substance. Secondly, the initial clinical features were nonspecific. Initial symptoms of nausea, vomiting, drowsiness, or mucosal burns are common to both paraquat and 2,4-D. Some of these symptoms may also mimic anticholinergic poisoning [2]. Subsequent development of renal failure and hypoxia are also well known with 2,4-D [6]. 

Acute paraquat self-poisoning is a significant problem in parts of Asia, the Pacific, and the Caribbean [7]. The most frequent routes of exposure to paraquat, either accidentally or intentionally, in humans and animals are following ingestion or through direct skin contact. If ingested, paraquat induces a burning sensation of the mouth and throat, followed by gastrointestinal irritation, subsequently resulting in abdominal pain, loss of appetite, nausea, vomiting, and diarrhoea. Direct contact with paraquat solutions or aerosol mists may cause skin burns and dermatitis. Paraquat splashed in the eyes can irritate, burn, and cause corneal damage and scarring of the eyes. Due to its low vapour pressure and the formation of large droplets, inhalation of paraquat spray used in the open environment has not been shown to cause any significant systemic toxicity; however, inhalational exposure to paraquat in confined spaces, such as a greenhouse, is known to be associated with fatal pulmonary disease. Irrespective of its route of administration in mammalian systems, paraquat is rapidly distributed in most tissues, with the highest concentration found in the lungs and kidneys. The compound accumulates slowly in the lungs via an energy dependent process. Excretion of paraquat, in its unchanged form, is biphasic, owing to lung accumulation and occurs largely in the urine and, to a limited extent, in the bile [1]. Poisoning with paraquat leads to both local and systemic effects. In an Indian series of 17 patients, the most common symptoms were vomiting (100%), followed by altered sensorium (59%), oral ulceration or dysphagia (53%), dyspnea (41%), or loose stools (24%) [4]. Systemic effects of paraquat are renal and hepatic failure, pulmonary edema and fibrosis, cardiac failure, shock, convulsions, and multiorgan failure. Involvement of lung in the form of diffuse alveolitis and subsequent pulmonary fibrosis is the hallmark of paraquat poisoning. Acute respiratory distress syndrome because of paraquat usually appears 24–48 h after ingestion [3]. In our patient, the cause of hypoxia was aspiration of blood. Cause of renal failure is multifactorial-hypovolemia, circulatory failure, septicaemia, and direct toxicity related to redox cycling [4]. 

Treatment involves removal of ingested paraquat by immediately induced emesis or by gastric lavage in a health care facility. Clay (Fuller's earth) and activated charcoal are effective adsorbents. Administer repeated doses of 60 gm of activated charcoal by gastric tube every two hours for three or four doses. Supplemental oxygen should be withheld unless the pO_2_ is less than 70 mmHg because oxygen may contribute to the pulmonary damage which is mediated through lipid peroxidation [8]. Hemoperfusion using activated charcoal has been shown to decrease paraquat level, but data to support survival benefit in humans is insufficient [4, 9]. It is only effective if initiated within 4 h of ingestion, as peak paraquat concentration in the lung is achieved in 5–7 h [4]. Hemodialysis is used as a support of acute renal failure, but it does not increase clearance of the substance as it is rapidly distributed to the lungs and other organs [4]. Immunosuppression with combination of cyclophosphamide and methylprednisolone was shown to be beneficial in moderate-to-severe cases by prevention of ongoing inflammation [5]. Since the principal biochemical mechanism for lung damage is initiated by oxygen-free radicals produced by peroxidation, clinicians have tried a number of antioxidant treatments in the hope that they might interfere with the process. Unfortunately, none of the studied treatments, including controlled hypoxia, superoxide dismutase, vitamins C and E, N-acetylcysteine, desferrioxamine, and nitrous oxide, has been proven to be effective [1, 10].

 Prognosis in paraquat poisoning is largely dependent on the amount of paraquat absorbed. According to our knowledge these are first cases of paraquat poisoning reported from the state of Rajasthan, India. These cases are reported to highlight the high mortality rate associated with paraquat poisoning in spite of advances in treatment and supportive care.

## 4. Conclusion

 There is no specific antidote available for paraquat poisoning. It is important to establish the diagnosis early and to pursue aggressive decontamination and prevention of further absorption. Increased awareness of the clinician and availability of the laboratory diagnostic methods will definitely help in successful management of paraquat poisoning.
